# State of the Art in Capsule-Based Dry Powder Inhalers: Deagglomeration Techniques and the Consequences for Formulation Aerosolization

**DOI:** 10.3390/pharmaceutics14061185

**Published:** 2022-05-31

**Authors:** Roman Groß, Kai Berkenfeld, Christoph Schulte, Anselm Ebert, Sunita Sule, Ameet Sule, Alf Lamprecht

**Affiliations:** 1 Laboratory of Pharmaceutical Technology and Biopharmaceutics, Institute of Pharmacy, University of Bonn, Gerhard-Domagk-Street 3, 53121 Bonn, Germany; rogr@uni-bonn.de (R.G.); kaib@uni-bonn.de (K.B.); 2 Presspart GmbH & Co. KG, Am Meilenstein 8–19, 34431 Marsberg, Germany; christoph.schulte@presspart.com (C.S.); anselm.ebert@presspart.com (A.E.); 3 Presspart Manufacturing Ltd., Whitebirk Industrial Estate, Blackburn BB1 5RF, UK; sunita.sule@presspart.com (S.S.); ameet.sule@presspart.com (A.S.)

**Keywords:** capsule-based dry powder inhaler, capsule oscillation movement, high-performance device, binary blends, spray-dried particle formulation

## Abstract

Commercially available dry powder inhalers (DPIs) are usually devices in a fixed combination with the intended formulation, and a change in medication by the physician often forces the patient to use a different device, requiring the patient to relearn how to use it, resulting in lower adherence and inadequate therapy. To investigate whether DPIs can achieve successful outcomes regardless of the formulation and flow rate used, a novel DPI and two commercially available devices were compared in vitro for their deagglomeration behavior for different binary blends and a spray-dried particle formulation. The results demonstrate that the novel device achieved the highest fine particle fraction (FPF) regardless of the formulations tested. In the binary mixtures tested, the highest emitted fraction was obtained by shaking out the powder due to the oscillating motion of the capsule in the novel device during actuation. For DPIs with high intrinsic resistance to airflow, similar FPFs were obtained with the respective DPI and formulation, regardless of the applied flow rate. Additionally, the development and use of binary blends of spray-dried APIs and carrier particles may result in high FPF and overcome disadvantages of spray-dried particles, such as high powder retention in the capsule.

## 1. Introduction

Dry powder inhalers (DPIs) are widely used for the therapy of lung diseases such as asthma, chronic obstructive pulmonary disease (COPD), or bacterial infections [1,2]. To achieve a sufficient therapeutic effect by deposition of the powder in the lower respiratory tract, the powder particles should have an aerodynamic diameter of <5 µm [3]. For this purpose, special manufacturing processes such as jet-milling of the active pharmaceutical ingredient (API) are used in the pharmaceutical industry to produce micronized particles in the inhalable size range [4,5,6]. However, increasing the total particle surface area by this technology often results in very cohesive particles that have poor aerosolization efficiency and flowability [7]. To overcome this problem and produce flowable and deagglomerable powder formulations, various formulation techniques are used, such as mixing the jet-milled particles with large carrier particles (e.g., lactose) to form interactive blends [8,9]. This method of formulation is preferred in the pharmaceutical industry due to the resulting high storage stability of the crystalline APIs [10]. In the case of particle engineered approaches, such as spray-dried particle formulations, the device should overcome the cohesive particle interactions during inhalation [11]. Apart from the formulation’s characteristics, it is known that the success of therapy with passive breath-actuated DPIs depends mainly on the physiological conditions of the patient and the inhalation profiles generated [12]. Inhalation performed with insufficient respiratory force and duration results in an unintentionally low emitted dose with insufficient powder deagglomeration and thus insufficient therapeutic success [13,14]. Since a COPD patient cannot achieve the same inspirational flow profile as a healthy person, this leads to decreased deposition of the powder in the lungs. To counteract this problem, high intrinsic resistance devices are being developed that should deliver the same amount of aerosol regardless of airway resistance and applied inhalation flow rate [2,15,16]. In addition to the aforementioned inhalation properties of the patient, the delivered powder fraction and the resulting aerodynamic properties of the aerosol are also related to the device properties, which include the opening mechanism of the capsule being used, the movement of the capsule (vibration, rotation, shaking), and the interaction between the powder, the capsule, and the inhaler wall [17]. The size and position of the holes pierced in the capsule also influence the aerosol properties [18].

Since the success of inhalation depends on many factors, as described, marketed delivery systems are designed as combination products consisting of the formulation and the DPI to minimize the number of potential sources of error. The formulation and the device are designed to work together to achieve a satisfactory therapeutic effect [19,20]. This results in a marketed formulation being prescribed by the physician only with the inhaler intended for it and patients having to relearn how to use a different inhaler when they change medications, which could affect treatment adherence [21]. Since different inhalers result in different deagglomeration of the same powder, developing and marketing a generic device is challenging.

**Figure 1:**
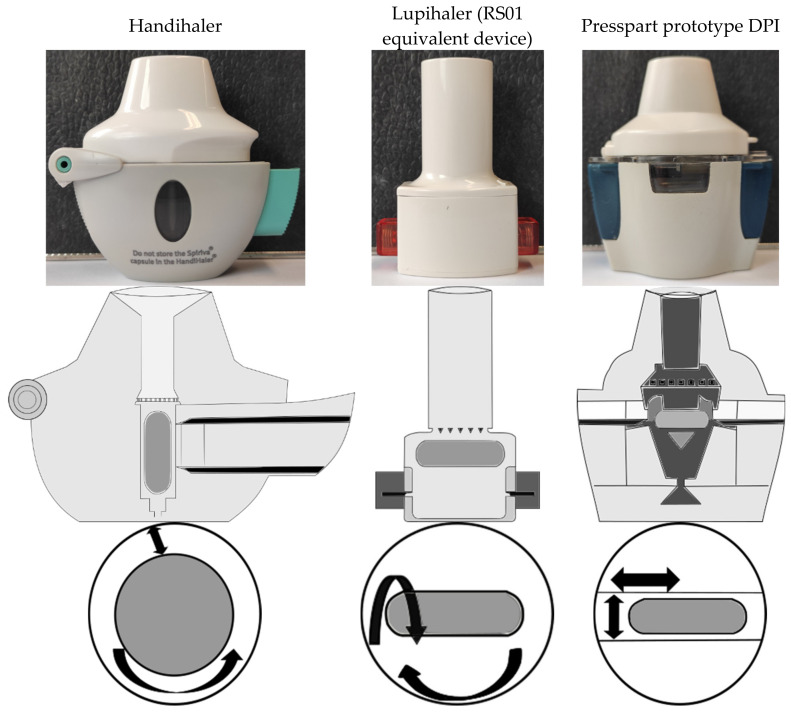
Schematic representation of the tested dry powder inhalers (DPIs) and the possible capsule movements that can be achieved in the various DPIs during inhalation. Modified from [22,23].

To investigate what the current market looks like, the study compared three capsule-based DPIs. Due to geometry and airflow, each unit has a different capsule motion and consequently a different mechanism for deagglomerating the powder. The study was designed to show which deagglomeration unit provides the highest fine particle fraction (FPF_TD/EF_ = fraction of particles with an aerodynamic diameter < 5 µm of the total dose/emitted fraction) regardless of the formulation tested and actuation conditions used. To determine the influence of capsule movement on powder ejection and the number of particles that can potentially reach the lungs, devices with axial capsule vibration (Handihaler^®^ (Boehringer Ingelheim, Ingelheim am Rhein, Germany)), capsule rotation (Lupihaler^®^ (Lupin Limited, Mumbai, India)) = RS01 equivalent device), and oscillating capsule movement (Presspart prototype DPI = PP-DPI) were compared (Figure 1) [22,23]. While the Handihaler^®^ and Lupihaler^®^ DPI are marketed devices that are well-known and extensively described, the Presspart prototype DPI is a novel capsule-based device. In order to analyze the “applicability of the devices for different formulations”, a series of drug formulations developed with different formulation techniques were tested. While the marketed formulation Cyclocaps^®^ (PB Pharma GmbH, Meerbusch, Germany) is an interactive blend of micronized albuterol sulfate and alpha lactose monohydrate, a spray-dried rifampicin formulation was also tested [1,24]. To demonstrate the potential use of carrier particles for spray-dried API particles, a spray-dried batch of amoxicillin was mixed with Inhalac 251^®^ (MEGGLE GmbH & Co. KG, Wasserburg am Inn, Germany) (ratio 1:24) in a further step to form a binary mixture, which was aerosolized using the above inhalers. To demonstrate the potentially flow-profile-independent deagglomeration behavior of the selected DPIs for the tested formulations, 50 L/min was selected as the low flow rate and 100 L/min as the high flow rate. These settings were also chosen because they closely approximate the actual flow rates of the low (Lupihaler) or high intrinsic resistance (PP-DPI, Handihaler) devices used in this study at a pressure drop of 4. To analyze the deagglomeration behavior of the devices for each formulation, in addition to the relative powder deposition in each stage, the FPF_TD/EF_ was compared.

## 2. Materials and Methods

### 2.1. Materials

The albuterol sulfate–alpha lactose monohydrate formulation (Cyclocaps^®^) (PB Pharma GmbH, Meerbusch, Germany) was purchased from a pharmacy. For spray-drying, rifampicin and amoxicillin were ordered from TCI (Tokyo, Japan). Except for water, which was purified in-house (Merck-Millipore Biocel A10, Burlington, MA, USA), all other solvents were HPLC grade. Inhalac 251^®^ was a kind gift from MEGGLE GmbH & Co. KG (Wasserburg am Inn, Germany). The Lupihaler^®^ devices (Lupin Limited, Mumbai, India) were purchased from a pharmacy in India. The Handihaler^®^ and Presspart prototype DPI inhalers were gifts from Boehringer Ingelheim (Ingelheim am Rhein, Germany) and H&T Presspart (Blackburn, United Kingdom), respectively.

### 2.2. Spray-Drying of the Rifampicin and Amoxicillin APIs

Rifampicin was spray-dried as described in [24]. Briefly summarized, the drug was suspended in ethanol (38 mg/mL) and sonicated in an ultrasonic bath (Typ DT 106, Bandelin electronic, Berlin, Germany) under controlled temperature conditions (25 °C) for 10 min. To keep the temperature constant during the water change, a thermostat (DC 10, Haake Technik GmbH, Vreden, Germany) was used. A B-290 spray-dryer equipped with a high-performance cyclone, a B-296 dehumidification unit, a B-295 (all Buchi, Flawil, Switzerland) inert loop, and an anemometer (AF89-AD1AA13C0AA, Fluid components Intl. San Marcos, CA, USA) was used for spray-drying under inert atmosphere (N_2_)_._ A modified three-fluid nozzle (Buchi, inner channel blocked) was used to atomize the suspension. During the entire manufacturing process, the suspension was constantly stirred.

For spray-drying of amoxicillin, the same spray-drying equipment was used. Prior to the spray process, 2 g of the API was dissolved in 185 mL MeOH, and the solution obtained was spray-dried (Table S1).

### 2.3. Mixing the Amoxicillin—Lactose Binary Blend

To generate the binary mixture, the spray-dried active ingredient was mixed with the carrier lactose monohydrate (Inhalac 251) in a ratio of 1:24. For this purpose, both materials were mixed in a sequential mixing process using a Turbula Mixer (Willy A. Bachofen, Muttenz, Schweiz) at 46 rpm for 5 min per mixing step until the mentioned mixing ratio was achieved.

### 2.4. Airflow Resistance of the Various DPIs

In order to measure the pressure drop across the devices and calculate the intrinsic resistance to airflow, each DPI was connected with a suitable adapter to a Dosage Unit Sampling Apparatus (DUSA), and that was connected with a flow meter (DFM 2000), a critical flow controller (CFC) (TPK 2100-R) and two vacuum pumps (HCP 5, all Copley Scientific Limited, Nottingham, United Kingdom). The pressure port of the DUSA was connected to the pressure port of the CFC. Measurements were made with a pressure drop from 1 to 8 kPa, and the specific resistance to airflow was calculated from the linear relationship between the square root of the pressure drop and the resulting flow rate (Figure S1).

### 2.5. Test Procedure for the Aerosol Classification with the Next-Generation Impactor

While the Cyclocaps capsules (albuterol sulfate–lactose) were purchased ready dosed, 5 mg of the rifampicin formulation and 30 mg of the amoxicillin–lactose blend formulation was filled into size 3 gelatine capsules. A Next-Generation Impactor (NGI) (Copley Scientific Limited, Nottingham, United Kingdom) was used to characterize the aerosol properties of the different formulations actuated with the different devices. Prior to the experiments, each cup was coated with a 1% glycerol-methanol (*m/v*) solution, and 15 mL of the diluent was placed in the preseparator unit. For analyzing the powder deagglomeration behavior of the DPIs, the particles were dissolved from each stage (capsule–MOC). While a water–methanol mixture (1:1% *v*/*v*) was used for albuterol sulfate and amoxicillin, rifampicin was dissolved in a solution of ascorbic acid in methanol (0.5% *m/v*). Flow rates of 50 and 100 L/min and an actuation volume of 4 L were used to compare the different devices in terms of aerosolization properties for the respective formulation. Unless otherwise reported, all experiments were performed in triplicate.

### 2.6. Scanning Electron Microscopy (SEM)

The samples were sputter-coated with gold for two cycles of two minutes each after being fixed with carbonaceous conductive paste on an aluminum sample holder. Subsequent imaging of the different formulations was performed in a high vacuum using a Hitachi SU-3500 SEM (Hitachi Ltd., Tokyo, Japan). While the magnification and working distance for each sample were set as needed and are reported in the SEM images, the accelerating voltage was set to 5 kV for all samples.

### 2.7. High-Performance Liquid Chromatography Analysis

Quantification of the active compounds deposited in the different NGI stages was performed by high-performance liquid chromatography (HPLC) analysis (Shimadzu, LC-2030C 3D Plus, Kyoto, Japan) using an RP18 column (Lichrospher 100 RP 18-5µ EC, 250 × 4.6 mm). To detect the APIs, the photodiode array detector was set to 337 nm for rifampicin, 275 nm for albuterol sulfate, and amoxicillin was detected at a wavelength of 230 nm. The mobile phase for both binary mixtures consisted of phosphate buffer/methanol (80:20 *v*/*v* (%)), and the buffer was adjusted to pH 3.0 in the case of albuterol sulfate and pH 4.0 for the amoxicillin. An isocratic flow of 1.4 mL/min (albuterol sulfate) or 1.0 mL/min (amoxicillin) was applied. For rifampicin, a flow rate of 1 mL/min of a mixture of a phosphate buffer pH 5.2/methanol/acetonitrile (33/50/17% *v*/*v*/*v*) was set. With the exception of rifampicin, which had a column temperature of 25 °C during quantification, the other active ingredients were analyzed at 40 °C. The limit of detection (LOD) and limit of quantification (LOQ) were calculated using the values of the intercepts and the slope of the calibration curve. For albuterol sulfate, the LOD and LOQ were determined to be 0.36 µg/mL and 1.08 µg/mL, whereas, for amoxicillin, the LOD and LOQ were calculated to be 0.97 µg/mL and 2.94 µg/mL. For rifampicin, the LOD was 0.30 µg/mL, and the LOQ was 0.90 µg/mL [24].

### 2.8. Statistics and Data Processing

To determine statistically significant differences in relative powder deposition in the different stages of the NGI (capsule–MOC) after actuation with different DPIs, results were compared using rank-sum ANOVA followed by Dunn’s test (*p* < 0.05) (Prism 8.0.2, GraphPad software). The choice of a non-parametric test was mainly based on the rather limited sample size of n = 3 in order to increase the robustness of the statistical decision, although all data were normally distributed. The NGI plots show the relative powder deposition in the different stages. The error bars indicate one standard deviation. Cumulative undersize plots from S1 to MOC were created and linearized by log transformation of the stage boundaries and probit transformation of the relative abundances. For calculating the fine particle fraction, a linear regression model was used (FPF_TD/EF_ (fraction of particles with an aerodynamic diameter < 5 µm of the total dose/emitted fraction)), as described in USP <601> [25].

## 3. Results

The Presspart prototype DPI can be classified into a top and a bottom unit. While the upper unit consists of a mouthpiece, a mesh, and a classifier, the lower unit is composed of the capsule chamber, the air inlet, and two buttons, each with a needle for piercing the capsule and the housing. Similar to the RS01 equivalent device, the capsule is placed horizontally in the capsule chamber and pierced from both sides, creating centered holes at the top and bottom of the capsule body. The air inlet is located below the capsule chamber, and a separator in the center of the inlet divides the airflow into two separate flow paths that flow past the top of the capsule or the side of the body. This results in an oscillating movement of the capsule with an axial rotation and vibration during actuation, which causes the powder to exit the capsule. After passing the classifier inlet, which sets the powder and the air stream into a cyclonic, dynamic airflow, a straight flow behavior is achieved by a subsequent mesh in combination with a vortex breaker in the mouthpiece until it exits the device (Figure S2).

**Figure 2:**
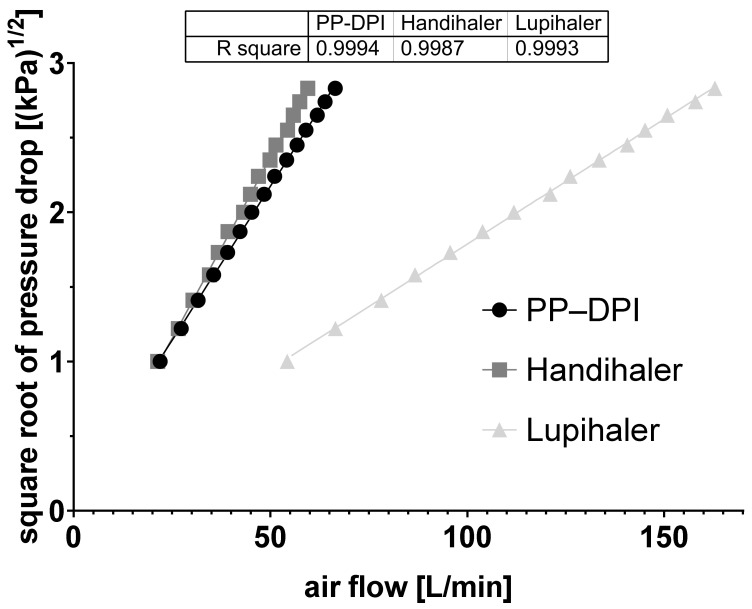
Relationship between the square root of pressure drop and the flow rate (L/min) across the novel (Presspart prototype dry powder inhaler (PP-DPI)) and both marketed DPIs (n = 3, mean ± SD).

To classify the new device as having low or high intrinsic resistance, the specific resistance to airflow was calculated from the linear relationship between ∆p and the resulting flow rate (L/min). The inspiratory resistance of the novel DPI was determined as 0.044 kPa^0.5^ L/min; the inspiratory flow rate at a pressure drop of 4 kPa was 45 L/min. The device can be categorized as a DPI with medium–high intrinsic resistance to airflow. The intrinsic resistance of 0.018 kPa^0.5^ L/min and the flow rate of 111 L/min at a pressure drop of 4 kPa, which was determined for the RS01 equivalent DPI, are in agreement with values found in the literature. This DPI is a low intrinsic resistance device. Due to the intrinsic resistance of 0.046 kPa^0.5^ L/min, the Handihaler can be classified as DPI with high intrinsic resistance to airflow (Figure 2) [16].

**Figure 3:**
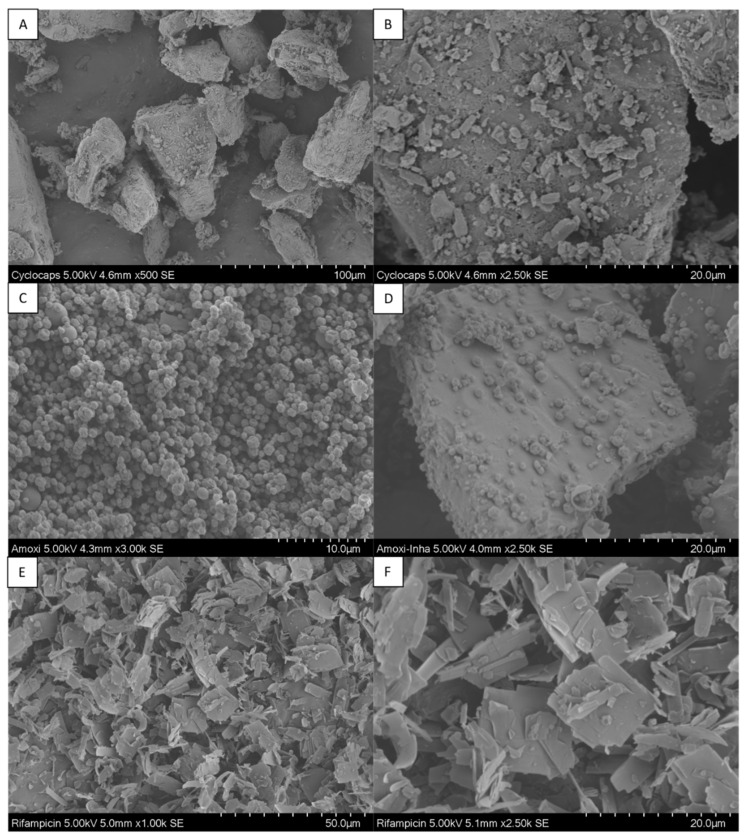
Scanning electron microscopy (SEM) images of the different formulations in different magnifications. (**A**,**B**) Cyclocaps [albuterol sulfate—lactose blend]; (**C**) amoxicillin; (**D**) amoxicillin–lactose blend; (**E**,**F**) rifampicin.

SEM pictures in Figure 3 show the size and shape of the particles of the formulations used. In the case of rifampicin, flocculent, platelet-shaped microparticles were seen. In the amoxicillin–lactose mixture, the spray-dried active ingredient, which had a spherical shape with a particle size in the lower micrometer range, was adsorbed onto the larger lactose carriers.

**Figure 4:**
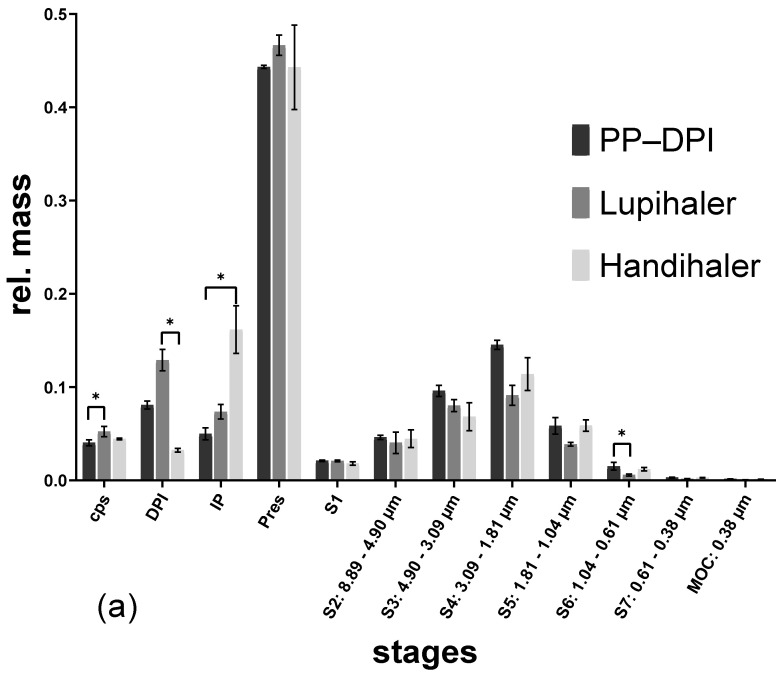

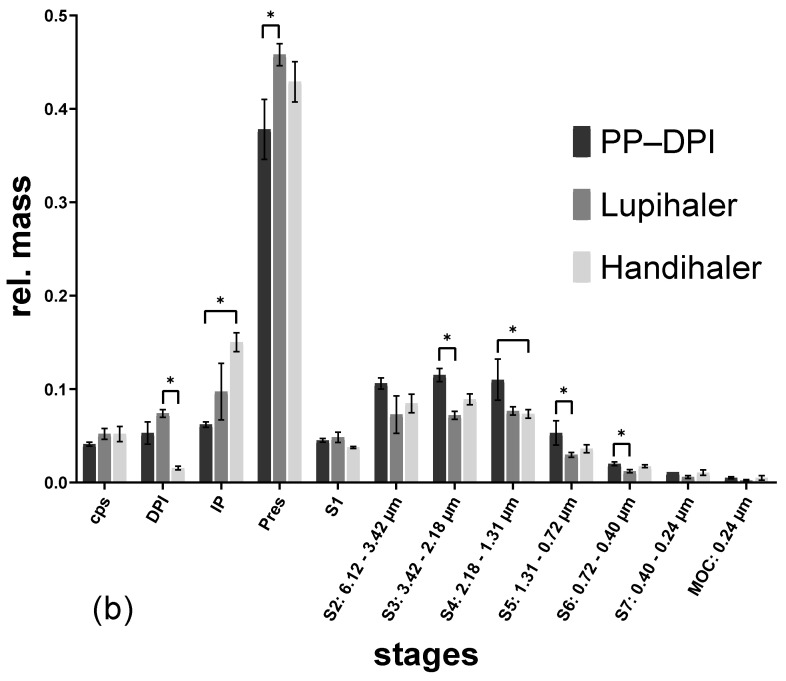
NGI results of the albuterol sulfate formulation actuated with different devices at different flow rates. (**a**) 50 L/min; (**b**) 100 L/min, (capsule = cps; dry powder inhaler = DPI; induction port = IP; preseparator = Pres), (* *p* < 0.05).

Testing the albuterol sulfate formulation with the different devices at 50 L/min resulted in a high powder deposition in the preseparator in every case (Figure 4a). While powder retention in the capsule was not affected by the different flow rates, there was less deposition in the device at the higher flow rate, regardless of the DPI used. The lower powder retention in the Handihaler, as well as the lower powder deposition in the induction port (IP) after using the Presspart prototype DPI, resulted in a higher FPF than with the Lupihaler (Table 1). While powder deposition in the preseparator was similar for each DPI used when operated at 50 L/min, operation at 100 L/min for the Presspart prototype DPI resulted in a lower deposition in the preseparator compared to the other two DPIs (Figure 4b).

**Figure 5:**
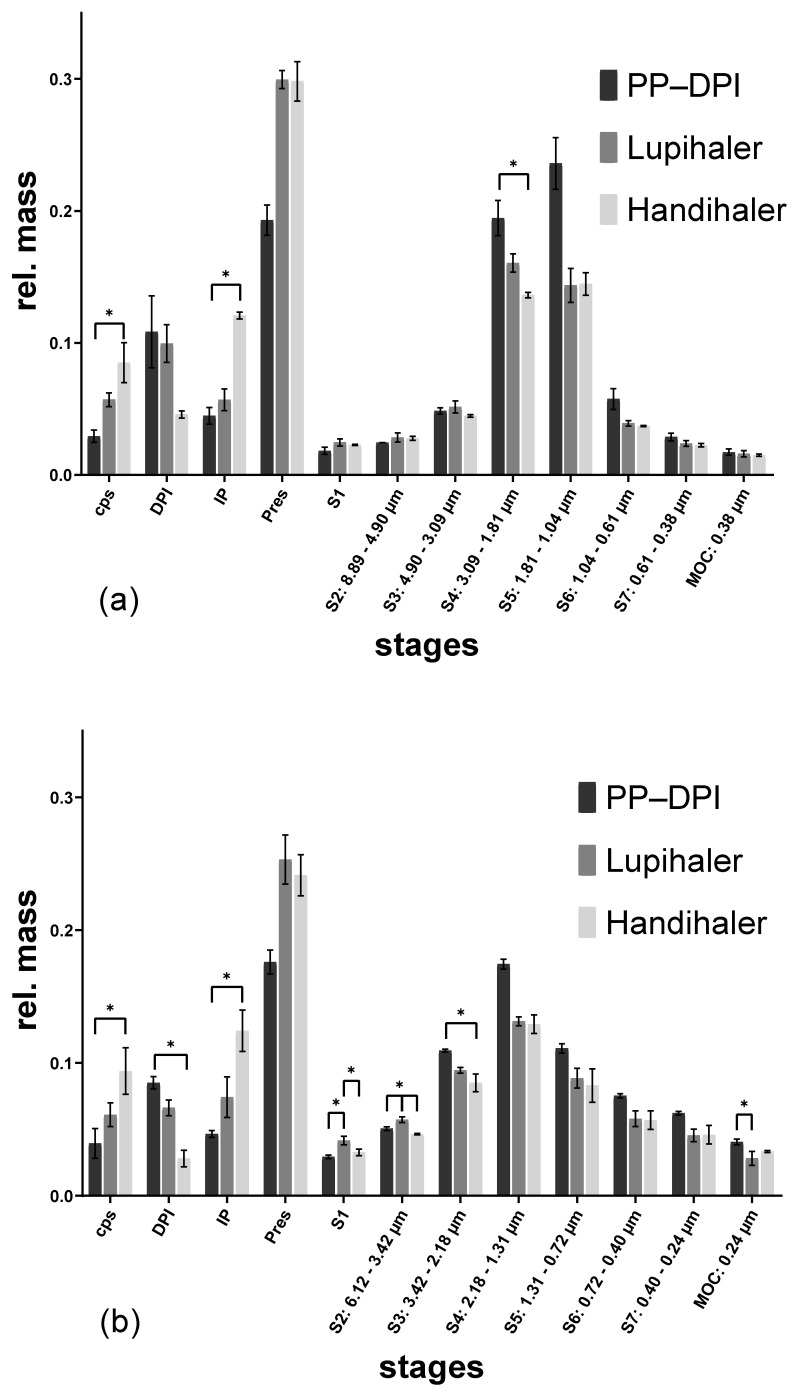
NGI results of the amoxicillin–lactose formulation actuated with different devices at different flow rates. (**a**) 50 L/min; (**b**) 100 L/min, (capsule = cps; dry powder inhaler = DPI; induction port = IP; preseparator = Pres), (* *p* < 0.05).

For the binary amoxicillin blend, the lowest powder retention in the capsule combined with the lowest powder deposition in the IP was observed after actuation with the Presspart prototype DPI, regardless of the flow rate applied (Figure 5). Comparing the influence of the inhalation airflow (50, 100 L/min) on the aerosolization of the powder actuated with the same device, no differences could be observed in the stages (capsule to preseparator). These resulted in similar FPFs of the emitted- or the total dose for both flow rates. While the Handihaler and the Lupihaler showed similar deagglomeration behavior for this formulation, with the exception of powder deposition within the capsule, the device, and the IP, the Presspart prototype DPI achieved the highest fine particle fraction (Table 1).

**Figure 6:**
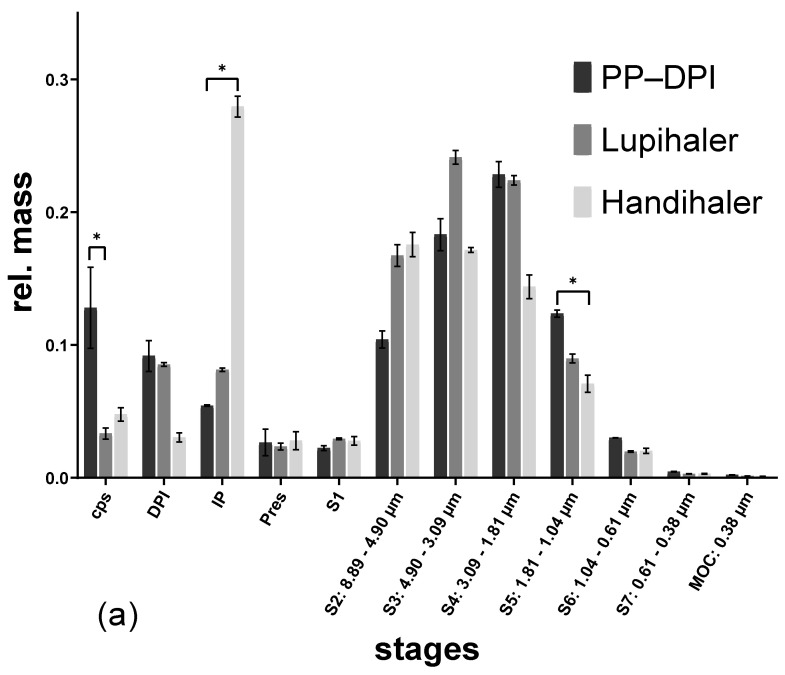

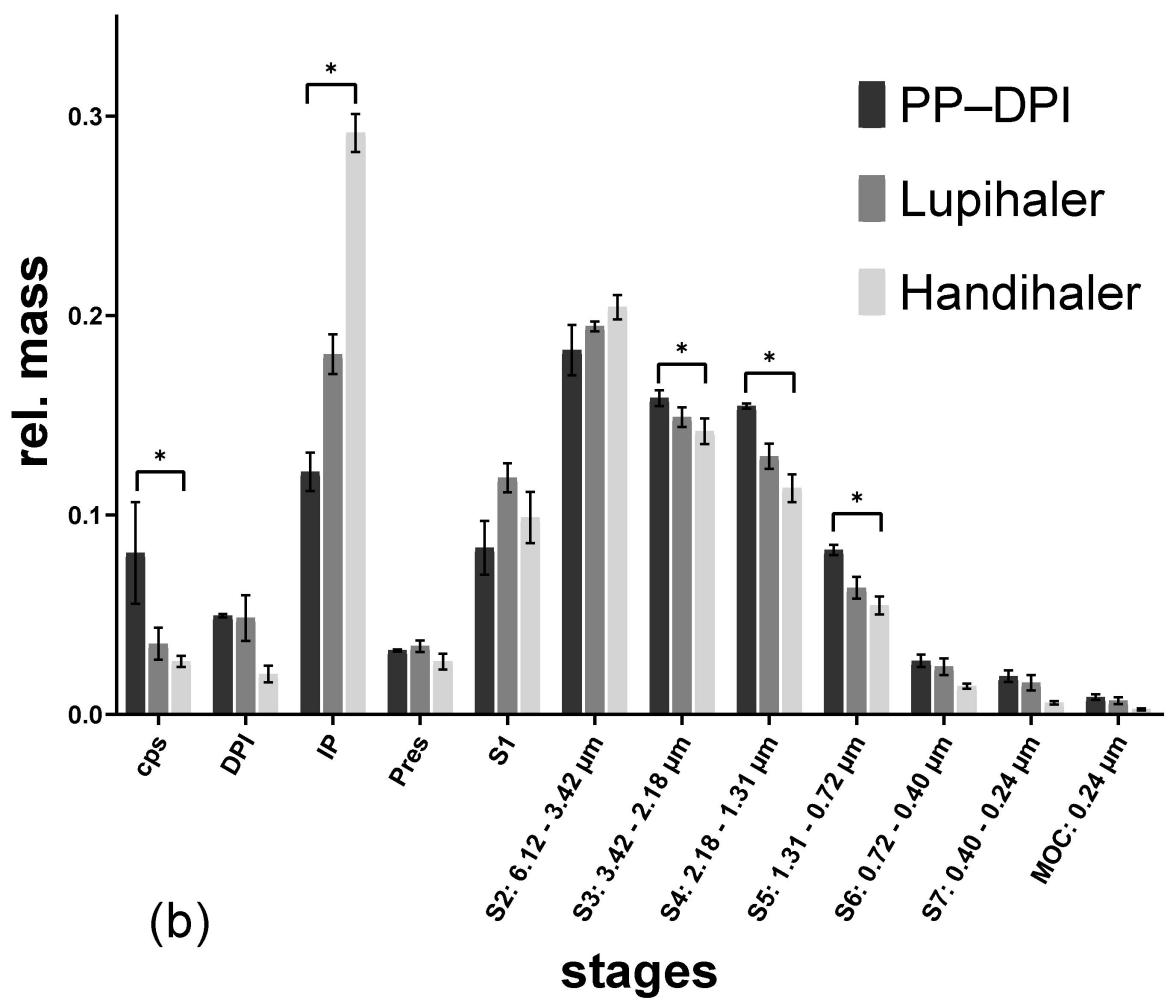
NGI results of the spray-dried rifampicin formulation actuated with different devices at different flow rates. (**a**) 50 L/min; (**b**) 100 L/min, (capsule = cps; dry powder inhaler = DPI; induction port = IP; preseparator = Pres), (* *p* < 0.05).

The results in Figure 6 show that rifampicin powder retention in the capsule was the highest when using the Presspart prototype DPI. This powder retention was independent of the flow rate applied. Of all the devices tested, the Handihaler had the lowest powder deposition in the DPI but also the highest powder deposition in the IP. Compared to the other units tested, this DPI exhibited similar powder deposition in the various stages of the NGI at both flow rates. This is underpinned by the consistent FPF_EF/TD_ (Table 1). Due to the high powder deposition in the IP, a lower FPF was achieved compared to that of the Lupihaler or Presspart prototype DPI.

While the Handihaler and Lupihaler showed similar deagglomeration behavior for the binary mixtures tested, resulting in similar powder deposition on S1–MOC, differences were evident for rifampicin between all devices tested. When comparing the powder deposition on the mentioned stages for the tested flow rate of 50 L/min, it is noticeable that with the Handihaler, the highest powder deposition was observed in stages S2–S3. While a high amount of API was deposited on S2–S4 when using the Lupihaler device, the Presspart Prototype DPI achieved a high powder deposition on the stages S4 and S5 (Figure 6a). At 100 L/min, a high amount of powder was deposited on S2–S4 for each DPI used (Figure 6b). Instead of the high powder deposition in the preseparator observed for both binary mixtures, regardless of the device used and the flow rate applied, a higher powder deposition in the IP was observed when actuating the rifampicin formulation.

At the tested flow rate of 50 L/min, the highest FPF_EF/TD_ was achieved with the rifampicin formulation, regardless of the DPI used. Due to the high powder retention in the capsule when using the Presspart prototype DPI, the FPF_TD_ was similar to that of the Lupihaler. The results of the 100 L/min data set showed a decrease in FPF for this formulation, which is due to the higher powder deposition in the IP, so the amoxicillin–lactose mixture had the best deagglomeration properties (Table 1).

## 4. Discussion

Since there are currently insufficient data on different DPIs and their ability to aerosolize different formulations not developed for the particular DPI, the idea arose to conduct a comparative study showing the advantages and disadvantages of selected DPIs in terms of deagglomeration and aerosolization of different drug formulations developed using different manufacturing techniques. These results could be used to derive state of the art in DPI development and indicate which deagglomeration mechanism offers potential for future development. This knowledge could be used for future commercialization of generic DPIs. To compare the different deagglomeration mechanisms, which are also influenced by the different movements of the capsule, a new capsule-based DPI with oscillating capsule movement was tested in addition to the two known commercially available DPIs (Handihaler, Lupihaler).

Classifying the novel device as a DPI with low or high intrinsic resistance to airflow and determining the pressure drop as a function of flow rate is an important parameter for verifying test conditions for conducting future studies. Due to the designed geometry resulting in intrinsic resistance of 0.044 kPa^0.5^ L/min and a flow rate of 45 L/min at a pressure drop of 4 kPa, the new capsule-based DPI can be classified as a device with medium–high intrinsic resistance to airflow. Based on the studies described in the introduction and the published results for this class of DPIs, the novel device should deliver the loaded powder uniformly to the patient regardless of the inhalation conditions, which could lead to higher treatment adherence in vivo [26]. With regard to the design of the novel DPI, a new approach was developed to eject the powder from the capsule by an oscillating movement. Considering that the capsule was pierced by two needles in each device tested, the influence of the number of needles and the resulting holes on powder output and deagglomeration was not part of this study. Nevertheless, previous studies have shown that the number and diameter of the needles, as well as the opening mechanism of the capsule, could have an influence on the aerosolization of the powder [18,22]. However, the current study focused on the influence of the device geometry, the resulting airflow through the DPI, and the resulting capsule movement.

For both binary mixtures, oscillatory capsule motion was shown to result in a higher emitted fraction than axial capsule vibration (Handihaler) or capsule rotation (Lupihaler), regardless of the flow rate applied. It can be concluded that for well-flowing binary blend formulations, the powder can be easily shaken out [4,6]. In the case of the rifampicin formulation, the inhalation force does not seem to be sufficient to eject the powder from the capsule, which could be due to the small particle size and the resulting increase in total surface area after spray-drying, leading to greater adhesion and cohesion forces of the powder and consequently greater adherence to the inside of the capsule wall or formation of aggregates [6]. In a previous study, it was also found that a platelet shape of particles reduces the flowability of the powder due to the resulting strong interactions between the individual particles [27]. Therefore, the oscillating motion of the capsule is not sufficient to overcome these interactions. Comparing the results of the albuterol sulfate or amoxicillin formulation obtained with the Lupihaler and the Handihaler device, it could be seen that similar powder deposition in the preseparator was observed in both cases. In addition, a higher API deposition in the IP was observed with the Handihaler compared to that of the Lupihaler. Both tendencies indicate that the deagglomeration of the powder and the detachment of the active ingredient from the carrier particles are insufficient, leading to the deposition of the mixture in the mentioned fractions. While for the albuterol sulfate formulation, the amount of drug deposited was independent of the flow rate applied, for the amoxicillin mixture, higher airflow resulted in less powder deposition in the preseparator, suggesting that this formulation was easier to deagglomerate. Comparing these observations with the results of these two formulations obtained with the Presspart prototype DPI, it was found that the amount of active ingredient decreased more in the two fractions mentioned, indicating a better deagglomeration behavior and possibly due to the high circulation speed in the classifier, which leads to a better detachment of the active ingredient from the carrier particles [28]. While no differences in powder deposition in the preseparator were observed for the albuterol sulfate formulation when the Lupihaler or Handihaler were used at different flow rates, a lower amount of drug was found using the Presspart prototype device when the flow rate was increased, which also reflects the better deagglomeration behavior of the new DPI for binary blends.

Despite the higher powder retention of the rifampicin formulation in the capsule, better deagglomeration of the emitted powder was observed with the Presspart prototype DPI than with the other two DPIs. The capsule movement described above, in combination with the device design, geometry, and airflow within the DPI, promotes powder deagglomeration so that the weak powder output from the capsule can be compensated.

Comparing the FPF_TD_, the Presspart prototype DPI achieved the highest FPF regardless of the formulation tested, and the flow rate applied, highlighting the functionality of the deagglomeration behavior for binary blends and spray-dried particle formulations. Since there were differences in powder deposition in the capsule–preseparator compartments, this resulted in differences in FPF_EF_ when comparing the two flow rates for a device and the respective formulation. While whether the powder remains in the capsule or DPI after inhalation is not important for therapeutic success, FPF_TD_ appears to be better suited to assess the independence of powder deagglomeration from inhalation conditions, as it describes the amount of powder of the loaded dose that can be adequately deagglomerated by the device and delivered to the lung in vitro, regardless of the inhalation conditions applied. Comparing the FPF_TD_ obtained for a formulation with the DPIs, it is noticeable that the two DPIs with high intrinsic resistance to the airflow achieved identical values at both flow rates, indicating that powder deagglomeration was not affected by the airflow.

From the device side, it can be summarized that the deagglomeration mechanism of the new DPI appears to offer an advantage over the known devices tested here in terms of powder deagglomeration, regardless of whether the formulation is a binary mixture or a particle engineered formulation. Although a high FPF was always achieved under the tested conditions, further development of the oscillating capsule movement mechanism could lead to increased powder ejection, especially for spray-dried particles.

## 5. Conclusions

From this study, firstly, it can be concluded that progress is being made in the development of DPIs with respect to the aerosolization behavior of various formulations not designed for the specific device and that, for this reason, the DPI could be marketed in the future regardless of the formulation. The development of DPIs with device geometry that provides high intrinsic resistance to airflow could lead to powder aerosolization that is much more independent of inhalation conditions than is currently the case. Second, the development of binary mixtures consisting of spray-dried active ingredients and carrier particles could be an interesting approach for future formulations, as the advantages of both formulation techniques can be combined to increase the number of particles that have suitable aerodynamic particle properties to reach the lungs.

## Figures and Tables

**Table 1:** Fine particle fraction of the emitted (FPF_EF_) or total dose (FPF_TD_) for the different formulations (AS* = albuterol sulfate–actose binary blend; amoxi–lactose blend* = amoxicillin–lactose blend; rifampicin) tested with various devices.

Flow Rate	Formulation	PP-DPI		Lupihaler		Handihaler	
[L/min]		FPF_EF_[%]	FPF_TD_[%]	FPF_EF_[%]	FPF_TD_[%]	FPF_EF_[%]	FPF_TD_[%]
50	AS*	34.7 ± 2.1	30.5 ± 1.8	25.1 ± 1.6	20.6 ± 1.3	27.2 ± 1.3	25.2 ± 1.2
	Amoxi–lactose*	66.4 ± 2.1	57.2 ± 1.8	50.3 ± 2.0	42.4 ± 1.7	45.2 ± 1.8	39.3 ± 1.6
	Rifampicin	73.0 ± 3.2	56.9 ± 2.5	66.3 ± 5.5	58.4 ± 4.8	47.8 ± 4.2	44.1 ± 3.8
100	AS*	41.3 ± 2.4	37.4 ± 2.2	27.3 ± 1.6	23.9 ± 1.4	29.6 ± 2.0	27.6 ± 1.9
	Amoxi–lactose*	68.8 ± 1.2	60.3 ± 1.1	55.2 ± 1.1	48.1 ± 1.0	52.5 ± 1.0	46.1 ± 0.9
	Rifampicin	63.5 ± 4.2	55.2 ± 3.6	53.7 ± 4.2	49.2 ± 3.8	46.6 ± 4.1	44.4 ± 3.9

## Data Availability

All data are contained within this article.

## Supplementary Materials

The following supporting information can be downloaded at: https://www.mdpi.com/article/10.3390/pharmaceutics14061185/s1, Table S1: Parameters used for spray-drying the rifampicin or amoxicillin particle formulations; Figure S1: Schematic diagram of the setup for determining the airflow resistance of the various dry powder inhalers (DPIs), (P1 = Pressure Port1); Figure S2: Exploded-view drawing of the Presspart prototype dry powder inhaler (PP-DPI).
