# Supplementary material for: State of the Art in Capsule-Based Dry Powder Inhalers: Deagglomeration Techniques and the Consequences for Formulation Aerosolization
Source: Pharmaceutics. 2022 May 31;14(6):1185. doi: 10.3390/pharmaceutics14061185 (PMC9230934; doi:10.3390/pharmaceutics14061185)
Supplement: Supplementary file 1 [file pharmaceutics-14-01185-s001.zip › pharmaceutics-1703663-supplementary.pdf]

pharmaceutics

MDPI

Article

Supplementary Materials: State of the Art in Capsule-Based Dry Powder Inhalers: Deagglomeration Techniques and the Consequences for Formulation Aerosolization

Roman Groß, Kai Berkenfeld, Christoph Schulte, Anselm Ebert, Sunita Sule, Ameet Sule and Alf Lamprecht

Table S1. Parameters used for spray-drying the rifampicin or amoxicillin particle formulations.

Formulation	Rifampicin	Amoxicillin
Feed rate [mL/min]	7.4	7.4
Spray gas flow [L/h]	375	601
Outlet temperature [°C]	60	85

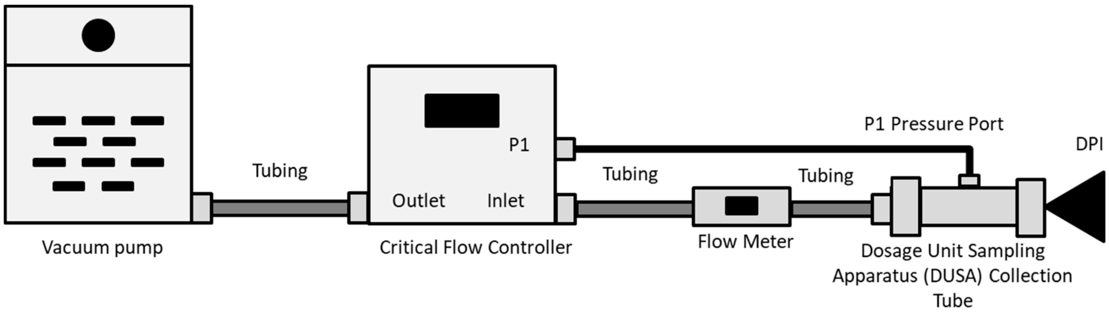

Figure S1. Schematic diagram of the setup for determining the airflow resistance of the various dry powder inhalers (DPIs), (P1 = Pressure Port1).

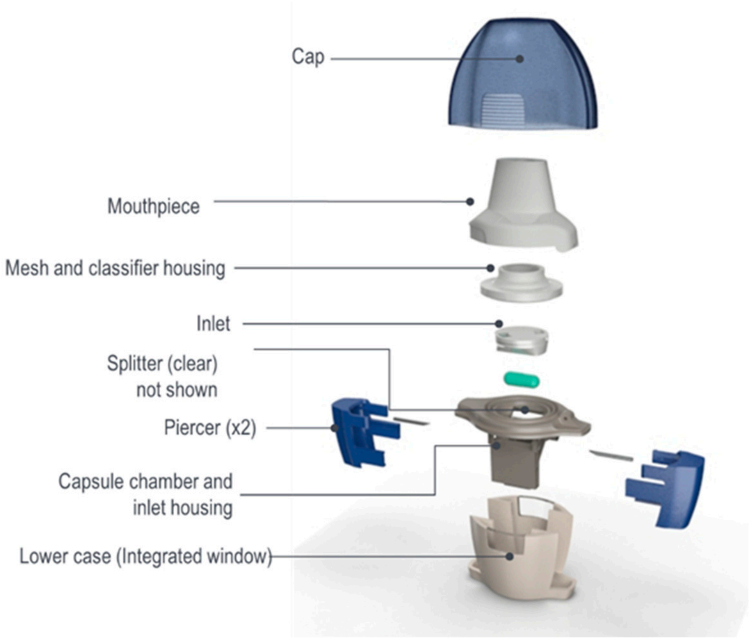

Figure S2. Exploded-view drawing of the Presspart prototype dry powder inhaler (PP-DPI).
